# An association between chronic life stressors prior to diagnosis of breast cancer

**DOI:** 10.17179/excli2021-4005

**Published:** 2021-08-31

**Authors:** Nathália de Sousa-Pereira, Mayara Bocchi, Caroline Yukari Motoori-Fernandes, Bruna Karina Banin-Hirata, Luiz Gustavo Piccoli de Melo, Karen Brajão de Oliveira, Glauco Akelinghton Freire Vitiello, Carlos Eduardo Coral de Oliveira, Clodoaldo Zago Campos, Marla Karine Amarante, Maria Angelica Ehara Watanabe

**Affiliations:** 1 Laboratory of DNA Polymorphisms and Immunology, Department of Pathological Sciences, Biological Sciences Center, Londrina State University, Paraná, Brazil; 2 Laboratory of Immunogenetics, Department of Basic Health Sciences, Biological Sciences Center, Maringa State University, Paraná, Brazil; 3 Department of Clinical Medicine, Health Sciences Center, State University of Londrina, Paraná, Brazil; 4 Laboratory of Molecular Genetics and Immunology, Department of Pathological Sciences, Biological Sciences Center, Londrina State University, Paraná, Brazil; 5 Department of Medicine, Pontifical Catholic University of Paraná, Londrina, Brazil

**Keywords:** breast carcinoma, oncology, risk factor, stress, chronic

## Abstract

The clinical course of breast cancer (BC) and survival depend on a wide range of risk factors. From the psychosomatic point of view, BC is one of the most studied type of cancer but there is no evidence available for this relation. Therefore, in the present study we evaluate the impact of chronic life stressors in BC patients. A total of 100 BC patients were invited to participate in an interview, when information about social parameters and emotional changes in the period prior to diagnosis were collected. The emotional changes were evaluated by the Holmes and Rahe's Stress Scale, which analyzes the difficulty required for a person to readjust to society after significant changes in their life. Clinicopathological parameters were obtained from the medical records. For all data, the level of significance adopted was p <0.05. It was observed that 55.2 % of the patients have a medium and 13.8 % were at high risk for disease development related to stressful events in the period prior to the BC diagnosis. The highest stress levels were presented by separated, divorced, or widowed patients compared to married (p <0.01) and single (p = 0.037) patients. The high-risk (HR) group had a lower proportion of positivity for estrogen receptor when compared to the low (LR) and moderate risk (MR) groups (p= 0.001). In addition, a binary logistic regression analysis was performed, and we found that the relationship between the estrogen receptor and the HR of chronic stress was independently associated with the histological type of BC and lymph nodes involvement. The relationship of stressful life experiences and BC is not well established, so our study collaborates with the literature to demonstrate the importance of stress as a factor associated with the development of BC.

## Introduction

Breast cancer (BC) is the most common malignant tumor in women, excluding cases of non-melanoma skin cancer. The estimated global incidence of BC was 2.3 million new cases in 2020 (Sung et al., 2021[34]). An estimated 66,280 new cases of BC are expected in Brazil for each year of the 2020-2022 triennium (INCA, 2019[15]). 

BC has high clinical, morphological and biological heterogeneity and is associated with different gene expression profiles, enabling the identification of distinct molecular subtypes, with prognostic factors and specific therapeutic targets (Cirqueira et al., 2011[5]). This molecular classification is made using the following markers in the clinical routine: estrogen receptors (ER), progesterone receptors (PR), overexpression of type 2 human epidermal growth factor receptor (HER2) and the cellular proliferation index Ki-67. Thus, from the gene expression profiles of these markers, four molecular subtypes were initially identified: Luminal-A (LA; ER/PR+ HER2-), Luminal-B (LB; ER/PR+ HER2+), HER2-enriched (HER2; ER- PR- HER2+) and triple-negative (TN; ER- PR- HER2-) (Cirqueira et al., 2011[5]; Perou et al., 2000[26]; Sorlie et al., 2001[32]).

Despite having a relatively good prognosis, when diagnosed in the early stages and treated in a timely manner, BC is detected in more advanced stages in low- and middle-income countries, significantly reducing patient survival (INCA, 2017[14]). The clinical course of BC and overall survival vary for each patient and depend on a number of risk factors, such as age, family history, early menarche, late menopause, advanced age in the first pregnancy, nulliparity and hormone replacement therapy (Ban and Godellas, 2014[2]; Brewer et al., 2017[4]; Horn et al., 2013[13]; Siegel et al., 2017[31]). Lifestyle, such as excessive use of alcohol and a high-fat diet, were identified as important risk factors (Jung et al., 2016[16]; Makarem et al., 2013[21]).

Since ancient times, there has been a belief that psychological factors, such as traumatic emotional experiences, could affect the susceptibility and course of a disease (O'Leary, 1990[25]). One of the main explanations for this phenomenon would be the theory of allostatic overload/bankruptcy. Allostasis is related to the maintenance of an intensely complex and dynamic internal balance of the different biological systems, functioning as “circuits interconnected in parallel”, which are activated concomitantly and can be influenced by both intrinsic and extrinsic stress factors (Goldstein and Kopin, 2007[10]; McEwen, 2000[23]; Sterling and Eyer, 1988[33]). When these stress modulation systems fail, that is, they suffer from a state of allostatic overload, they promote chronic activation of the physiological response to stress, such as, a hyperactivation of the hypothalamic-pituitary-adrenal axis, increased pro-inflammatory cytokines and a state of chronic hypercortisolism. Changes can generate irreparable damage to the individual and lead to the development of diseases related to chronic stress, such as cancer, psychiatric disorders and cardiovascular diseases (McEwen, 1998[22]; McEwen and Gianaros, 2010[24]).

BC is one of the most studied types of cancer from the psychosomatic point of view, mainly because it is one of the most prevalent in the female population. Cormanique et al. (2015[6]), using the Self-Reporting Questionnaire (SRQ-20) scale, developed by the World Health Organization (WHO) to screen for psychiatric disorders, showed that women with a previous history of chronic stress has a higher prevalence of the HER2 BC subtype. Dourado et al. (2018[8]) showed an association between stressful life events after BC diagnosis, using the Holmes and Rahe Stress Scale, and the development of metastasis. However, Santos et al. (2009[29]), through a meta-analysis, concluded that stress-producing life events would not be associated with risk of BC.

Although there is much discussion and controversial results about the role of stressful life events and cancer, little is known about the impact that past chronic life stressors have on determining BC and its respective molecular subtypes. Therefore, the objective of the present study was to evaluate the association of previous chronic stressors to the BC diagnosis, assessed by the Holmes and Rahe Stress scale, and their respective molecular subtypes in women monitored at Londrina Cancer Hospital (LCH), Paraná, Brazil.

## Methods

### Sample selection

The present study was approved by the Ethics Committee of the Londrina State University, Paraná, Brazil, agreeing with the National Commission of Ethics in Research (CAAE: 68744617.0.0000.5231). The BC patients were invited, randomly, to participate in this project during the clinical care in LCH and the informed consent form was signed by all patients prior to data collection.

### Data collection

A total of 100 BC patients participated in an interview after medical care at LCH, with the objective of collecting information about social parameters, such as marital status, ethnicity, number of children and job occupation. In addition, patients were asked about events that marked emotional changes in a period prior to BC diagnosis, which were evaluated by the Holmes and Rahe Stress Scale (Holmes and Rahe, 1967[12]). This scale analyzes the difficulty required for the person to readjust to society after significant changes in their life, which generate emotional distress leading to various diseases. The instrument consists of a list of 42 events, such as the death of a spouse or a close relative, divorce, marital separation, professional changes, among others. The instrument measures the intensity and duration of the time needed to adapt to a life event and is based on the concept that any change is considered a stressful factor. Each event has a score given by the authors of the instrument, ranging from 11 to 100 points. 

During the interview, the patients reported several situations that caused them some emotional change and all of them could be identified in the events presented by the Holmes and Rahe Stress scale. For the statistical analyses, the final score of each patient was considered, which consisted of the sum of the points of each event reported by them. The Holmes and Rahe Stress scale were categorized into 3 groups for risk of disease development associated with chronic stress levels: low risk (LR) (final score lower than 150), medium risk (MR) (final score 150-299) and high risk (HR) (final score greater than 300).

The clinicopathological parameters were obtained from the medical records of these patients and the immunohistochemical analysis for the HER2, ER, PR and Ki-67 markers was performed at the Laboratory of Clinical Pathology of the LHC, following a standard protocol (Hammond et al., 2010[11]; Wolff et al., 2013[35]). Of the patients analyzed, 95 were classified according to the expression of the markers in specific molecular BC subtypes.

### Statistical analysis

The data obtained in the interviews were used to evaluate the distribution and relation of social parameters and clinicopathological parameters with the levels of chronic stress before BC diagnosis. Initially, univariate analyzes were performed and, subsequently, multivariate analyzes to rule out possible confounding factors, respecting the criteria for each statistical test. For all data the significance level adopted was p <0.05. The SPSS 22.0 (Chicago, Illinois, USA) statistical program was used for all analyzes.

## Results

All women were interviewed and were stratified according to the expression of hormone receptors, HER2 overexpression and Ki-67 cell proliferation index in molecular subtypes. We observed that 45 % of patients were classified as Luminal-B, 42 % as Luminal-A, 4 % as HER2 and 4 % as TN. In addition, 5 patients could not be classified according to molecular subtypes due to lack of data in medical records. Distribution of social and clinicopathological characteristics, such as number of children, marital status, ethnicity, age, among others are shown in Table 1(Tab. 1).

To analyze the involvement of chronic stressors in the period prior to BC diagnosis, the patients reported whether any daily events would have led to an emotional change in them. Patients who did not feel comfortable talking about their emotional feelings or stressful events (n = 13) were excluded from the analysis, thus a total of 87 patients were analyzed. It was observed that 31 % of the patients were at a LR, 55.2 % were at a MR and 13.8 % were at HR for developing disease due to the stress levels in the period prior to BC diagnosis. 

We observed that most patients (69 %) reported the death of a spouse, family member or close friend as an important fact in their emotional change before BC diagnosis. However, no statistical difference was observed between the risk groups (p = 0.089, data not shown).

The next step was to associate the groups at risk of developing diseases associated with chronic stress with the sociodemographic parameters (Table 2(Tab. 2)). There was an association between the patients' marital status and the chronic stress groups prior to diagnosis (p = 0.012). Based on this result, women were classified according to their marital status and the medians of the final scores obtained by the Holmes and Rahe stress scale for each group were compared. It was observed that the highest levels of stress were presented by separated, divorced or widowed patients compared to married (p <0.01) and single (p = 0.037) patients.

Finally, we evaluated the association between the risk groups and the clinicopathological parameters (Table 3(Tab. 3)). A lower proportion of ER positive was observed in women in the HR group when compared to the LR and MR groups. A binary logistic regression analysis was performed using the ER as a dependent variable and the histological types, lymph nodes involvement and the HR group as independent variables (Table 4(Tab. 4)). The relationship between the ER and the HR of chronic stress (Holmes and Rahe ≥ 300) was independent of the histological type and lymph nodes involvement. Marital status was not included due to high collinearity with HR of chronic stress on this scale used.

## Discussion

In the present study, it was observed an association between patients' marital status and risk groups for developing diseases based on stress levels, with the highest stress levels presented by the separated, divorced or widowed patients compared to married and single patients. Furthermore, women in the HR group had a lower proportion of positivity for ER when compared to the LR and MR groups. There are no data prior to this study in the literature demonstrating the association of stress levels with clinicopathological parameters of BC.

Marital status influences quality of life, a fact that was demonstrated in a study in which elderly people who were married or living in a stable union had better psychological quality of life compared to widowed, single, divorced elderly people (Roncon et al., 2015[28]). The involvement of marital status with the risk of developing BC has been reported in several studies in the literature. A prospective study by Lillberg et al. (2003[20]) demonstrated a two-fold increase in the risk of developing BC in a group of Finnish women after divorce and separation process. A similar result was observed by a case-control study of Li et al. (2016[19]), in which Chinese women with marital status considered disharmonious are 1.16 times more likely to develop BC. However, other studies demonstrate the protective factor of marital status (married, separated, divorced and widows) in relation to the development of BC (Ewertz, 1986[9]; Kvikstad et al., 1994[18]; Lillberg et al., 2003[20]).

An interesting association has been demonstrated by Schoemaker et al. (2016[30]), in a cohort of 113,000 UK women, where the risk of developing BC with ER negativity was higher in women after divorce or separation. The authors pointed out that the association was restricted to premenopausal women, consistent with literature data showing that these women are more likely to develop ER negative breast tumors compared to postmenopausal women. Although the sample number in our study is lower, we demonstrated an association between women who had low levels of stress according to the Holmes and Rahe Stress Scale (classified in the LR group) and ER positivity. Since the highest scores on the scale are given to major events in the separation or divorce process, we can conclude that most women who made up this group were single and married.

Of the patients analyzed in our study, 55.2 % had a MR and 13.8 % had HR of developing a disease based on the stress levels, according to the scale used. In a study by Barbosa and dos Santos (2012[3]), using another instrument to detect stress levels (Lipp Adult Stress Symptom Inventory), it was observed that 60 % of BC patients were diagnosed with stress. Of these patients, 40 % were in the resistance, 15 % in the near-exhaustion and 5 % in the exhaustion phase.

From our patients', we observed that most (69 %) of them described death of a spouse or close family member (mother, father, child, among others) as a stressful event within a variable time of 1 year before the BC diagnosis. BC studies have demonstrated the importance of this event in cancer patients. The risk of developing BC was higher in women who described the death of their husband, family member or close friend as a stressful event in their life (Kruk, 2012[17]; Lillberg et al., 2003[20]).

The relationship between stress and the immune system has been discussed in the literature. Several studies have linked chronic stress to a weakened immune system (Barbosa and dos Santos, 2012[3]). Stress has been associated with suppression of immune function and there is evidence that it may promote the initiation of some cancers, especially those associated with DNA viruses, retroviral insertion near an oncogene, and other viruses (Reiche et al., 2004[27]). In this context BC has been associated with several viral infections, such as human papillomavirus (HPV), Epstein-Barr virus (EBV) and more recently the human breast tumor virus (MMTV-like) (Amarante and Watanabe, 2009[1]; de Sousa Pereira et al., 2020[7]). Prolonged unemployment, depression and bereavement are stressors that seem to produce a diminished immune response, with cases of prolonged immunosuppression. More than two decades ago, O'Leary (1990[25]) reviewed empirical evidence linking emotional processes to immune function in humans.

## Conclusion

In this study, we can verify the association between chronic life stressors prior to BC diagnosis with marital status and positivity for ER, demonstrating the social and clinicopathological panorama of BC patients attended at the LCH, Paraná, Brazil. Despite being considered an important factor in the development and progression of cancer, the relationship between stressful life experiences and BC is not well established. Therefore, our research collaborates with the literature in an attempt to confirm the importance of stress as a factor associated with BC development.

## Acknowledgement

The authors would like to thank all the volunteers who made this study possible. This study was supported by the Conselho Nacional de Desenvolvimento Científico e Tecnológico (CNPq) and the Fundação Araucária do Paraná, Extension Program “University Without Borders”, of the Secretary of State for Science, Technology and Higher Education and Pró-Reitoria de Extensão from Londrina State University.

## Conflict of interest

The authors report no conflict of interest related to this research.

## Figures and Tables

**Table 1:**
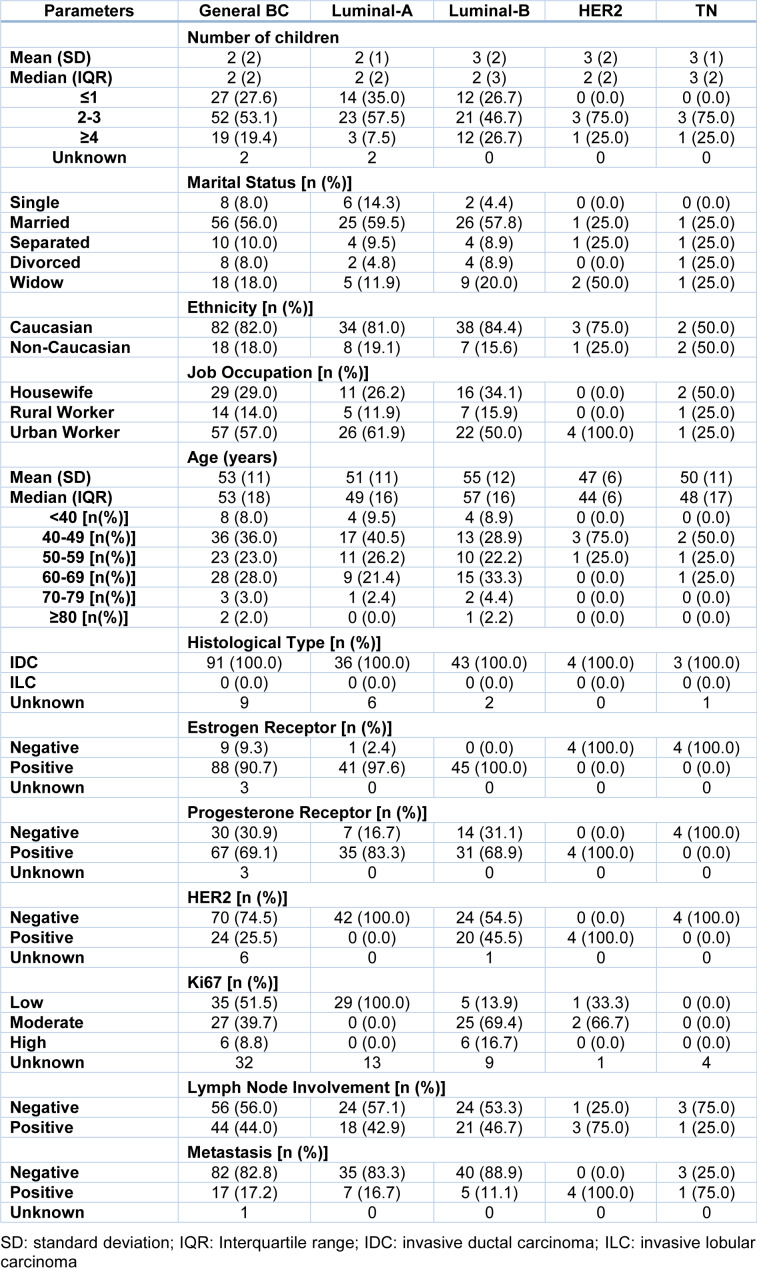
Patients' social and clinicopathological characteristics

**Table 2:**
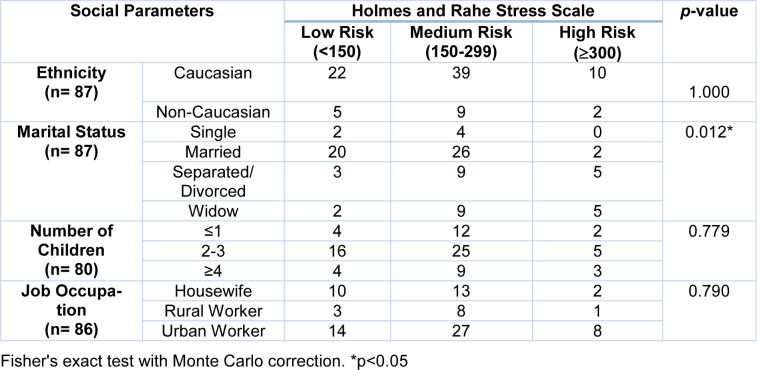
Association analysis between groups of risk of disease development associated with chronic stress levels and social parameters

**Table 3:**
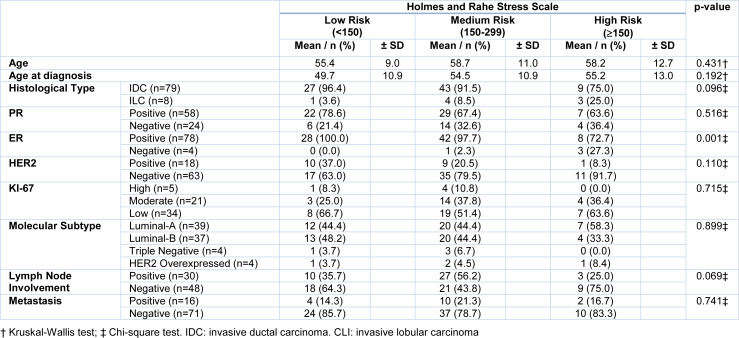
Analysis of the association between groups of risk of disease development associated with chronic stress levels with clinicopathological parameters in patients

**Table 4:**
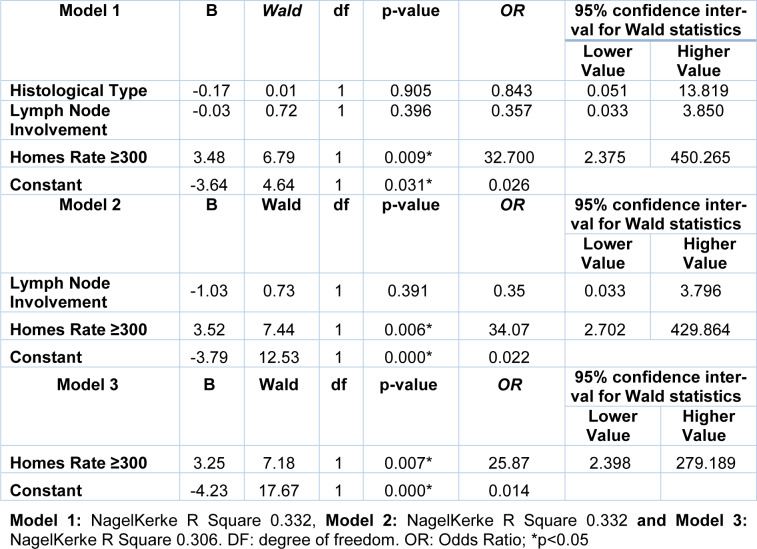
Binary logistic regression analysis by the backward method using the estrogen receptor as dependent variable
